# Supplementary material for: Development of chemokine network inhibitors using combinatorial saturation mutagenesis
Source: Commun Biol. 2025 Apr 3;8:549. doi: 10.1038/s42003-025-07778-6 (PMC11969024; doi:10.1038/s42003-025-07778-6)
Supplement: Supplementary file 4 — Supplementary Data 2 [file 42003_2025_7778_MOESM4_ESM.zip › SupplementaryData3/scripts/xAF3.tar!/./script/analysis/JK/plots/ligbondplot.pdf]

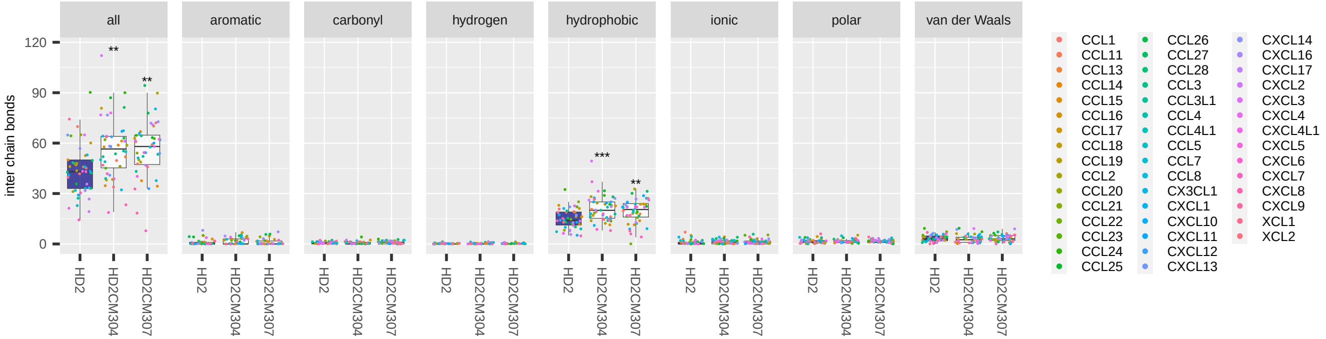

all
aromatic
carbonyl
hydrogen
hydrophobic
ionic
polar
van der Waals
inter chain bonds
120
90
60
30
0
**
**
***
**
HD2
HD2CM304
HD2CM307
CCL1
CCL11
CCL13
CCL14
CCL15
CCL16
CCL17
CCL18
CCL19
CCL2
CCL20
CCL21
CCL22
CCL23
CCL24
CCL25
CCL26
CCL27
CCL28
CCL3
CCL3L1
CCL4
CCL4L1
CCL5
CCL7
CCL8
CX3CL1
CXCL1
CXCL10
CXCL11
CXCL12
CXCL13
CXCL14
CXCL16
CXCL17
CXCL2
CXCL3
CXCL4
CXCL4L1
CXCL5
CXCL6
CXCL7
CXCL8
CXCL9
XCL1
XCL2
